# Hierarchical Classification of Event-Related Potentials for the Recognition of Gender Differences in the Attention Task

**DOI:** 10.3390/e23111547

**Published:** 2021-11-20

**Authors:** Karina Maciejewska, Wojciech Froelich

**Affiliations:** 1 Institute of Biomedical Engineering, Faculty of Science and Technology, University of Silesia in Katowice, 75 Pulku Piechoty 1a Street, 41-500 Chorzow, Poland; 2 Institute of Computer Science, Faculty of Science and Technology, University of Silesia in Katowice, Bedzinska 39 Street, 41-205 Sosnowiec, Poland; wojciech.froelich@us.edu.pl

**Keywords:** gender identification, event related potentials, ERP signal classification, data mining

## Abstract

Research on the functioning of human cognition has been a crucial problem studied for years. Electroencephalography (EEG) classification methods may serve as a precious tool for understanding the temporal dynamics of human brain activity, and the purpose of such an approach is to increase the statistical power of the differences between conditions that are too weak to be detected using standard EEG methods. Following that line of research, in this paper, we focus on recognizing gender differences in the functioning of the human brain in the attention task. For that purpose, we gathered, analyzed, and finally classified event-related potentials (ERPs). We propose a hierarchical approach, in which the electrophysiological signal preprocessing is combined with the classification method, enriched with a segmentation step, which creates a full line of electrophysiological signal classification during an attention task. This approach allowed us to detect differences between men and women in the P3 waveform, an ERP component related to attention, which were not observed using standard ERP analysis. The results provide evidence for the high effectiveness of the proposed method, which outperformed a traditional statistical analysis approach. This is a step towards understanding neuronal differences between men’s and women’s brains during cognition, aiming to reduce the misdiagnosis and adverse side effects in underrepresented women groups in health and biomedical research.

## 1. Introduction

Recently, the importance of recognizing gender differences in health and biomedical research, including neuroscience, has become a significant matter of concern, prompting new policies to be implemented at the funding agencies (Canadian Institutes of Health Research, European Commission, US National Institutes of Health or German Research Foundation) to supervise gender analysis [1,2,3,4].

In recent decades, experiments in biomedical research were carried out without considering gender, resulting in more significant health risks for women due to their under-representation in the preclinical, clinical studies, and drug trials. Higher rates of misdiagnosis and adverse side effects from drug treatment were also more common for women. Acknowledging gender-specific functional differences is of great importance in the clinical research of brain disorders such as autism, conduct disorder, attention-deficit hyperactivity disorder (ADHD), schizophrenia, dyslexia, stuttering, Tourette’s syndrome, major depression, anxiety, panic disorders, obsessive-compulsive disorder (OCD), posttraumatic stress disorder (PTSD), bulimia, migraines, multiple sclerosis (MS), myasthenia gravis, and Alzheimer’s disease.

In this paper, we address this crucial research problem of cognitive neuroscience. We examine whether gender differences in human cognition can be recognized while performing the attention task. We used event-related potentials (ERPs) measured while performing attention tasks by a cohort of women and men to accomplish that. We used that data as an input to the data mining method we propose in this paper. The method proposed here combines practical and theoretical efforts to solve the underlying stated problem. As interdisciplinary research, this work contributes to both neuroscience and computer science. The contribution of this paper should be considered from empirical and theoretical perspectives:

- We propose a carefully designed original experimentation procedure enabling the acquisition of electroencephalography (EEG) signals in the human attention task. In contrast to many EEG-based classification studies, where the EEG datasets were acquired from open databases, we designed and ran a neurophysiological experiment. Thus we ensured complete control of the possible confounds that are unknown when using public databases, allowing us to detect gender differences using the proposed data mining technique;
- The theoretical contribution is the data mining method, which relies on the hierarchical segmentation and classification of ERPs. In particular, we transform the preprocessed ERPs to a multivariate time series. The time-series underlies segmentation, enabling the construction of a chain of classifiers yielding the targeted gender classification;

To the best of our knowledge, this paper is the first study proposing data mining recognition of gender differences using the ERP signal from an attention task.

The remainder of this paper is organized as follows. In Section 2, by presenting a literature review on the addressed problem, we motivate and prove the originality of the research undertaken in this paper. To deal with the addressed problem, we formalize it in Section 3.1. Then, in Section 3.2, we go to the detailed presentation of the proposed method. The results proving the high effectiveness of the proposed method are provided in Section 4. Section 5 concludes the paper.

## 2. Literature Review

ERPs are time-locked brain responses to stimuli, measured using EEG. They are scalp-recorded signatures of neural processes generated by groups of neurons activated during cognitive functions. The ERP component is defined as the scalp-recorded activity generated by a specific neural or psychological process, which produces defined polarity, latency, and scalp distribution, and is sensitive to experimental manipulations [5]. Traditionally, in ERP studies, amplitude and latency (i.e., the time interval between stimulus onset and the waveform) are measured, and these measures are compared between conditions. However, averaging the ERP trials to the stimuli onset across all the trials, i.e., the presentations of the stimuli during an examination, introduces a jitter that lowers the effect size and standard methods may not be sensitive enough [5].

Due to large individual differences and the small effect size of gender differences, there is still no consensus in the currently available literature regarding gender-related differences in the ERP waveforms [6,7,8,9,10,11,12,13,14,15]. Therefore, there is a need to search for more sensitive tools that are able to capture gender-related differences among healthy participants. It is necessary to explore computational approaches in neuroscience to present results that are both accurate and more robust. Consequently, neuroscientists have recently started searching for different machine learning classification algorithms as a tool for EEG-based decoding for studying the neural coding of human cognition, as they may increase both the stability of ERP data and classification accuracy.

ERP classification can be used to determine whether studied groups of subjects differ from each other. The purpose of using classification techniques for EEG signal analysis is to increase the statistical power of the differences between conditions that are too weak to be detected using standard ERP analytical methods. A classifier is then trained to distinguish between ERP time series from one group and the ERP time series from another group, and it then separates the two classes. Therefore, EEG classification methods may serve as a valuable tool for studying the temporal dynamics of human brain activity, and this study was designed to that end. That means that the classification was performed on averaged ERPs to understand the nature of underlying signals. That is why in such applications, obtained accuracies are informative, even though they are not as high as observed in brain-computer interface (BCI) applications, where high accuracy is essential due to single-trial decoding for prediction.

However, classification techniques to analyze EEG signals for understanding are still a novelty. Even less has been done to classify ERPs related to attention, one of the most important cognitive processes. While most EEG-based classification studies rely on resting-state EEG recording, in this work we focused on ERPs in reaction to visual stimuli as a result of neuronal activity related to the cognitive process, allowing for the isolation of specific cognitive functions of the human brain. In an oddball attention task, a P3 component is observed, which is related to the number of attentional resources engaged during task performance and reflects stimulus classification speed, thus being a sensitive temporal measure of the neural activity underlying the processes of attention allocation and immediate memory [16,17].

Among all gender classification methods, EEG-based gender recognition is precious, since EEG captures cognitive processes, revealing the estimated gender-related differences in the brain. It has multiple advantages, such as: high accuracy, high permanence, inherent uniqueness, universality, and resistance to deception [18]. However, the number of studies focused on gender classification in cognition is very limited due to the small effect size of gender differences and the impossibility to use a within-subject study design. Some of the previous studies showed the reliability and potential trustworthiness for gender classification using EEG signal [19,20,21,22,23,24,25,26,27].

Hu et al. compared a combination of four entropy feature sets, six single classifiers, and three ensemble algorithms as a method to identify gender based on resting-state EEG signal [20]. The authors found their results promising in providing a more efficient method for recognizing gender. Van Putten et al. explored deep learning for gender classification based on frequency features of the resting-state EEG [26]. Nguyen et al. [24] proposed a framework of automatic age and gender classification using EEG data from a person. The features were sent to a machine learning module, for example, support vector machine (SVM), to build age and gender models for that person. The experiments suggested that the paralinguistic features were very promising for this task. In another work of these authors [25], they proposed a framework based on parallel factors (PARAFAC), multilinear partial least squares (N-PLS) and SVM, which that can automatically classify age and gender using frequency features of the resting-state EEG data with eyes open and eyes closed. Li et al. [23] investigated the potential gender differences in resting-state EEG signals. Ghani [19] investigated gender classification of normal subjects based on their frontal resting-state EEG signals. Kaushik [22] used deep bi-directional long short term memory (BLSTM-LSTM) neural network to construct a hybrid learning framework for age and gender prediction based on resting-state EEG recording of subjects with closed eyes. Kaur et al. [21] presented an automatic age and gender prediction framework of users based on their neural signals captured during eyes-closed resting-state EEG. Wang [27] proposed a hybrid model for EEG-based gender recognition in the resting-state, which showed the potential applicability of the proposed approach and its ability to identify personal gender in an EEG-based biological recognition system.

However, these studies were based on resting-state EEG recordings, which do not capture gender-related differences in cognitive processes, such as attention, memory, language comprehension, or decision making. Specific cognitive tasks may achieve better performance due to more pronounced gender differences. There have been studies that applied EEG-based classification methods to different cognitive processes: anxiety [28], motor imaginary [29], working memory and spatial attention [30,31], emotions [32,33,34,35], or preference recognition in neuromarketing [36]. However, we found only two studies that investigated the gender-related difference in EEG during a cognitive task. De La Pava et al. [37] studied the gender differences present in an EEG-based emotion level classification system by means of simple K-nearest neighbor classifiers. The obtained results showed a gender-related difference for the valence dimension of the emotion scale in terms of classification performance. However, this work did not use the ERPs, which capture the dynamics of brain activity. Bilalpur et al. [38] studied gender and emotion recognition with ERPs and eye-tracking during emotional face processing. The authors concluded that gender differences were encoded best for anger and disgust.

In this paper, we use a careful and well-thought-out preprocessing pipeline, which is used in conventional ERP studies but not in the EEG-based classification studies (see [39] for a review showing that over 60% of the studies reviewed did not systematically remove EEG artifacts). In addition, using Target-Standard difference waveforms, i.e., subtracting ERPs to standard stimuli from ERPs to target stimuli, minimized potential confounds related to non-neural sources (e.g., physical properties of the stimuli or non-neural between-subject differences). This is a recommended approach in ERP studies that uses traditional methods but is not commonly used, especially not in EEG-based classification studies.

In our previous work, we examined ERPs in a standard visual P3 paradigm among healthy participants by means of an empirical and statistical approach to evaluate gender-related differences in ERPs [40]. A nonparametric cluster-based test showed significant differences in ERPs between men and women. In this paper, we present a new, hierarchical approach for more efficient gender recognition.

The novelty of our current approach lies in combining the neuronal signal preprocessing with the classification method, with a contribution of segmentation step, into a full line of electrophysiological signal analysis during the attention task to a better differentiation of the attention-related ERPs in men from ERPs in women.

## 3. Materials and Methods

Before going to the presentation of the proposed method, we specify the addressed problem.

### 3.1. Problem Specification

Let $p=[1,2,\ldots ,p_{max}]$ be an index of a person participating in an experiment, where $p_{max}\in Z$ is a parameter, the number of persons considered. From each *p*th person, we gather continuous measurements from *d* electrodes that are indexed by e=[1,2,…,d]. We assume $z_{e}^{p}\left(\tau \right)\in R$ is a single, real-valued measurement, gathered during the experiment from the *p*th person and *e*th electrode. By $\tau \in [\tau_{min},\tau_{max}]$ we denote real-time measured from the beginning of the measurement at which the measurement was taken, where $\tau_{min}=0$. The period of the experiment is determined by an interval $\left[\tau_{min},\tau_{max}\right]$, where $\tau_{min},\tau_{max}$ are the real-valued parameters.

By putting together all the measurements gathered at time τ from the *p*th person and *d* electrodes, we get a *d*-dimensional vector $Z^{p}\left(\tau \right)\in R^{d}$. A sequence $\left\{Z^{p}\left(\tau \right)\right\}$ of those vectors is gathered over the time of each recording.

Let us consider now a mapping between $\left\{Z^{p}\left(\tau \right)\right\}$ and the gender of the considered *p*th person. We denote that mapping as $M(\left\{Z^{p}\left(\tau \right)\right\})\to G$, where $G={\{}^{\prime }female^{\prime }{,}^{\prime }male^{\prime }\}$ is the set of class labels g∈G that are to be assigned to the sequence $\left\{Z^{p}\left(\tau \right)\right\}$. Note that under the above assumptions, the mapping *M* is a classifier identifying the gender of a given person.

For the sake of completeness of the introduced notation, we denote by $2^{Z(\tau )}$ a power-set of {Z(τ)}, i.e., a huge space of data sequences that can be potentially gathered from the experiments independent of the considered person.

Let us assume the gender of a given person is unknown, i.e., we do not have a classifier *M* available. Thus, we face a problem of discovering *M* and then making an assignment $g=M(\left\{Z^{p}\left(\tau \right)\right\})$, if possible, for each $\left\{Z^{p}\left(\tau \right)\right\}\in 2^{Z(\tau )}$. This is a problem we address.

### 3.2. The Proposed Method

The method proposed here covers the data acquisition process, data preprocessing, and classification. Let us first take a look at the proposed approach from a general perspective.

1. Acquisition of ERP signals. To cope with the stated problem, we discover *M* using a supervised type of learning, i.e., we induce *M* from sample data $\left\{S\in 2^{Z(\tau )}\right\}$ with the known classifications. Those sample data *S* are gathered through experiments. They are also used for the validation of the proposed approach;
2. Data preprocessing. We preprocess the gathered data to form a *d*-dimensional multi-variate time series. This step leads to a reduction of the considered data. Note that by the data preprocessing we replace the problem of discovering *M*, by looking for a classifier $M_{1}$ that deals with the produced multivariate time series instead of the raw data gathered from the experiments. Therefore, the quality of data pre-processing is pivotal for the reliability of the proposed approach;
3. Time series segmentation. We segment the previously produced time series in the time domain. Thus, we replace the problem of discovering $M_{1}$ by a more straightforward problem of constructing $M_{2}$ a classifier that, instead of dealing with the entire time series, classifies only a much shorter part (segment) of them. The issue that arises here and that we solve through computational experiments is the selection of the time segment that best suits the classification when combined with the classifier $M_{2}$;
4. Bottom-level classification. Finally, we classify the selected segment of the time series by combining the classifications of each vector contained in that segment. It means we construct $M_{2}$ by combining the classifications delivered by a standard, state-of-the-art classifier denoted here as $M_{3}$. The obstacle that we come across here is the selection of $M_{3}$.

In the above-described way, we create a hierarchy of data processing steps. In particular, we form a chain of classifiers $M\to M_{1}\to M_{2}\to M_{3}$. Thanks to that, we transform the addressed problem in a way that on its bottom level (classifier $M_{3}$) we are able to use a standard, state-of-the-art classifier known from the literature.

#### 3.2.1. Acquisition of EEG Signal

Twenty students participated in the experiment (23.1 ± 1.1 years, 10 women). All participants were right-handed, had normal color perception, normal (or corrected to normal) visual acuity, and normal blood pressure and body temperature at the time of the study. They were healthy, non-smokers, and had no history of neurological or psychological disorders. Information about their health conditions and lifestyles was collected via questionnaire. None of the participants had consumed alcohol, coffee, intoxicants, or energizing beverages (or other such substances) within 12 h prior to the study (based on the questionnaire), which could have had an impact on subjects’ cognition. Participants were also asked to get adequate rest, not to attend parties or other tiring events, and not to consume large amounts of alcohol the day before the examination.

The participants were seated in a comfortable chair in front of the computer screen at a distance of 1 m, in a dimmed room. A two-stimulus oddball paradigm, which included standard (frequent) and target (rare) stimuli, was used. The visual stimuli consisted of images of white and black geometric figures, presented in a randomized order in the center of a 19 inch LCD monitor. A black square on a white background was the target stimulus, and a white circle on a black background was the standard stimulus. The length of the square side and the diameter of the circle were 9 cm each. Participants were instructed to press a button when they saw the target stimulus, and to gaze at the center of the black screen during the inter-stimulus interval. The visual P3b potential was elicited in response to task-relevant target stimuli. The parameters of the stimuli were: 150 ms duration, 1000 ms inter-stimulus interval, 20% target and 80% standard stimulus probabilities. The total number of stimulus presentations was 300, including 240 standard and 60 target stimuli. The scenario of the experiment was created using Eevoke software (ANT Neuro, Hengelo, The Netherlands).

Continuous EEG was recorded from 32 Ag/AgCl electrodes embedded in an elastic Waveguard${}^{\mathrm{TM}}$ EEG cap (ANT Neuro, Hengelo, The Netherlands), using extended 10/20 EEG montage system with the AFz electrode as the ground electrode. Before the signal acquisition, participants’ skin was prepared for the examination. Everi (Spes Medica s.r.l., Genova, Italy) abrasive and conductive paste was used to clean the skin on the hairless areas of the scalp to remove dead skin and skin impurities before putting on the EEG cap. Then, the EEG cap was put on and OneStep Clear Gel (H + H Medizinprodukte GbR, Münster, Germany) was inserted into all the electrodes in order to provide contact between skin and electrodes. The impedance at each electrode site was kept below 5 kΩ. The signals were recorded using a common average reference. The EEG signal was collected by way of ANT Neuro (Hengelo, The Netherlands) amplifier (AMP-TRF40AB model) in DC with 20,000 amplification gain and 256 Hz sampling rate. No high-pass filter was applied during data acquisition. The acquisition and data pre-processing of EEG signals were performed according to the International Federation of Clinical Neurophysiology (IFCN) Guidelines for eliciting, recording, and quantifying mismatch negativity, P300, and N400 [41]. Advanced Source Analysis system ASA-Lab (ANT Neuro) with ASA v.4.8 software was used for the acquisition and offline data pre-processing.

In this study, an EEG signal was measured from each participant’s head during the attention task. During acquisition, the biological analog signal is converted into a discrete, digitized signal, where each data point (i.e., potential value in μV) is measured and saved every time step. This time step is the result of the sampling frequency $F_{s}$ [Hz = 1/s], which is the number of samples obtained in one second. Our data have been measured with 256 Hz, so the time step is 1/256 Hz = 0.00390625 s = 3.90625 ms. Therefore, the raw, continuous signal contains all the data points collected within the experiment, which is approximately 5 min. However, since our goal is to classify the EEG signal in the attention task, we have to perform pre-processing of the raw data. Aside from dealing with the artifacts, pre-processing contains epoching and averaging the epochs, which allows obtaining ERPs time-locked to the presented stimuli.

This way, we produce the sequence $\left\{Z^{p}\left(\tau \right)\right\}$. Note that the length of that sequence differs across the participants since there was an approximately 30 s additional time interval before the start and after the stop of the stimulation presentation of the attention task. This was done in order to allow the EEG signal to stabilize and for the subsequent data pre-processing. However, this difference does not influence the classification process because the classification was performed on the data epoched time-locked to the stimuli onset.

#### 3.2.2. Data Pre-Processing

**Figure 1:**
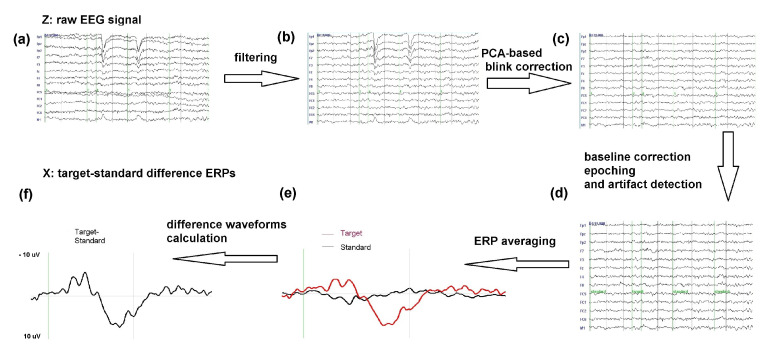
Event-related potentials (ERPs) signal pre-processing workflow: (**a**) raw electroencephalography (EEG) signal, (**b**) filtered EEG signal, (**c**) EEG signal after blink correction, (**d**) EEG signal after baseline correction, epoching and artifact detection, (**e**) averaged ERPs to target, and standard stimuli, (**f**) averaged ERP target-standard difference waveform. Please note the reversed polarity of the signal in this figure.

To obtain ERP from the raw EEG $\left\{Z^{p}\left(\tau \right)\right\}$, we introduce a function Φ representing data pre-processing. Using that function, we perform a transformation $\Phi (\left\{Z^{p}\left(\tau \right\}\right)\to \left\{X^{p}\left(t\right)\right\}$. This transformation is performed due to the fact that during the EEG measurement time $\tau \in [\tau_{min},\tau_{\max}]$, a set of visual stimuli were presented to the participants, divided into two categories: standard stimuli and target stimuli. Participants were asked to focus their attention on the target stimuli. Therefore, the task-related ERPs come from the transformed $X^{p}\left(t\right)$ dataset. Figure 1 shows the workflow of EEG pre-processing, which included data filtering, blink correction using PCA, baseline correction, epoching, artifact detection, ERP averaging, and ERP target-standard difference waveforms calculation.

In the data pre-processing step, the recorded EEG signal was first filtered using a non-causal Butterworth band-pass filter (with 0.01–30 Hz half-amplitude cutoff and 24 dB/oct slope). In order to correct for eye blinks artifacts, principal component analysis (PCA) was conducted. An alternative way to reduce artifacts is independent component analysis (ICA) [42,43,44], however, in order to maintain a full agreement to the previously presented results, we performed the classification on the same pre-processed data as the previous experiment. A signal with amplitude over ±75 μV was detected and removed from the analysis. After signal filtering and correcting for eye artifacts (see Figure 1a–c), for each participant, the continuous data has been epoched, i.e., epochs (time intervals) from −100 ms to 1000 ms time-locked to each of the 300 stimuli have been extracted (see Figure 1d). This means each epoch started 100 ms before the stimulus onset (baseline) and ended 1000 ms after the stimulus onset, resulting in 281 time points in each epoch. After baseline correction and detrending, all the epochs were averaged across all standard and target stimuli separately (see Figure 1d,e). The mean (± standard deviation) number of standard and target stimuli after data pre-processing were: 160 ± 32 and 40 ± 8 in women and 164 ± 51 and 40 ± 13 in men, respectively. Finally, in order to isolate the ERPs related to the task, the ERP difference waveforms were calculated by subtracting the averaged ERPs from the standard stimulus category from the averaged ERPs from the target stimulus category (target-standard difference waveforms, see Figure 1e,f).

Note that while performing the transformation $\Phi (\left\{Z^{p}\left(\tau \right\}\right)\to \left\{X^{p}\left(t\right)\right\}$, the initially considered continuous time scale is epoched, and the epochs are averaged across the presented stimuli. After that, it is represented by t∈T, where: $T=\{1,2,\ldots ,t_{\max}\}$. The period of the experiment measured in real-time $\left[\tau_{min},\tau_{\max}\right]$ is thus mapped to $\left[1,t_{\max}\right]$, where $t_{max}$ is a parameter common for all persons and experiments. Thus instead of dealing with time in a continuous scale τ, we consider time steps *t* within the epoch.

Thanks to the data pre-processing and the time discretization, which we describe in detail in the following section, the gathered data can be considered as multivariate time series. Specifically, data collected at time *t* from the *e*th the electrode of a particular *p*th person form a univariate time series $\left\{X_{e}^{p}\left(t\right)\right\}$. By collecting data from all electrodes, we get a multivariate time series. i.e., $\left\{X^{p}\left(t\right)\right\}=\left[\left\{X_{1}^{p}\left(t\right)\right\},\left\{X_{2}^{p}\left(t\right)\right\},\ldots ,\left\{X_{d}^{p}\left(t\right)\right\}\right]$, where t∈T. Thus, the time series $\left\{X^{p}\left(t\right)\right\}$ is composed of univariate time series $\left\{X_{e}^{p}\left(t\right)\right\}$.

#### 3.2.3. Data Segmentation

**Figure 2:**
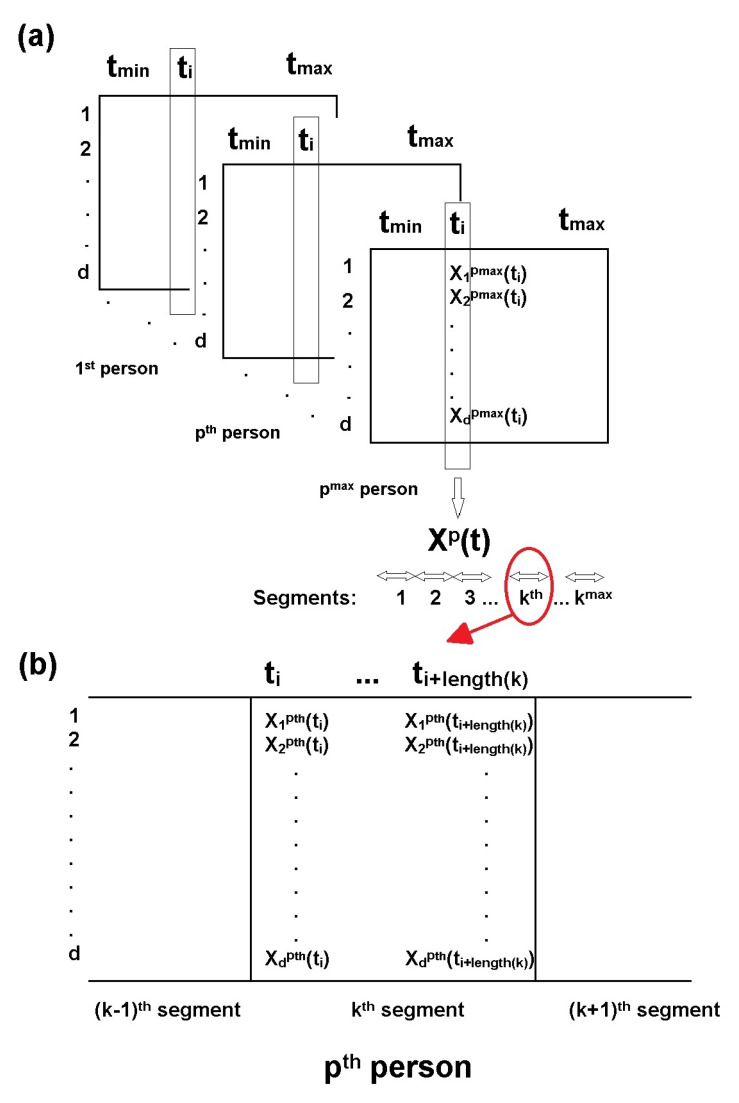
Epoched data specification (**a**) and segment specification (**b**). Panel (**a**) represents datasets for each person: from $t_{min}$ to $t_{max}$ timepoints and for d electrodes. Panel (**b**) represents the exemplary k-th segment.

After collecting the time series $\left\{X^{p}\left(t\right)\right\}$ we partition them into segments $\left\{W_{k}^{p}\left(t\right)\right\}\subset \left\{X^{p}\left(t\right)\right\}$, where $k=1,2,\ldots k_{\max}$ is an index of a segment, and $k_{max}$ is a number of those segments. Figure 2 presents the epoched data specification (panel a) and a segment specification (panel b).

We measure the length $\left|\{W_{k}^{p}\left(t\right)\}\right|$ of each segment as a number of vectors $X^{p}\left(t\right)\in \left\{W_{k}^{p}\left(t\right)\right\}$ contained in the *k*th time interval, corresponding to that segment.

Thanks to the performed segmentation, instead of classifying the entire time series $\left\{X^{p}\left(t\right)\right\}$, we use for that purpose a single, much shorter part of it, namely a segment $\left\{W_{k}^{p}\left(t\right)\right\}$. This way, we again reduce the amount of data that needs to be classified.

**Figure 3:**
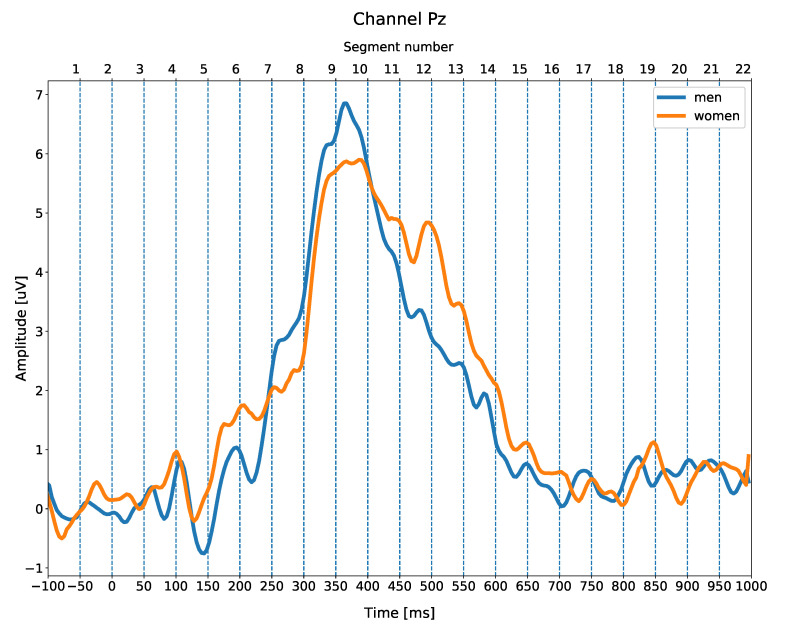
Grand averaged target-standard difference ERPs for men (blue) and women (orange) at an exemplification electrode site Pz (midline parietal). The bottom time scale represents the length of the epoch used for data averaging (−100 to 1000 ms with −100–0 ms baseline). The top time scale represents the number of time segments (1–22) corresponding to 50-ms time bins.

The result of the segmentation process is illustrated in Figure 3, which presents the grand averaged ERPs for men and women, i.e., the mean ERPs from each participant averaged across all the men and all the women, for a representative Pz electrode site (midline parietal).

The bottom time scale represents an epoch used for data averaging (i.e., −100 to 1000 ms time-locked to the stimulus presentation) with −100–0 ms serving as the baseline. The top time scale represents consecutive numbers of time segments (in the range 1-$k_{max}$), $k_{\max}=22$, which correspond to 50-ms time bins in the original time scale. For example, the segment k=10 covers time series related to the epoched data gathered in period t∈[350–400ms] measured between 350 ms and 400 ms after the stimuli onset.

Thanks to the segmentation, instead of using $M_{1}\left(\left\{X^{p}\left(t\right)\right\}\right)$, we are able to use a classifier $M_{2}\left(\left\{W_{k}^{p}\left(t\right)\right\}\right)$, for *k*th segment. The selection of that segment, i.e., the value of *k*, for which the classification of $\left\{W_{k}^{p}\left(t\right)\right\}$ leads to the best classification accuracy, is the goal of the computational experiments we perform at the experimental stage.

#### 3.2.4. Bottom-Level Classification of ERP Waveforms

Note that each segment $\left\{W_{k}^{p}\left(t\right)\right\}$ is a multivariate time-series, i.e., it contains a sequence of d-dimensional vectors $X^{p}\left(t\right)$ that have to be classified. To classify each of those vectors separately, we are able to use one of the standard, state-of-the-art classifiers. Here, we denote it as $M_{3}\left(X^{p}\left(t\right)\right)$.

More specifically, we employ $M_{3}$ to generate $g_{3}^{\prime }\left(t\right)=M_{3}\left(X^{p}\left(t\right)\right)$, where $g_{3}^{\prime }\left(t\right)$ denotes the classification delivered by the classifier $M_{3}$ for each $X^{p}\left(t\right)\in \left\{W_{k}^{p}\left(t\right)\right\}\}$. Note that this classification may not always be correct, i.e., $g_{3}^{\prime }\left(t\right)\neq g$.

Going backward in our hierarchy of classifiers, we need to produce the classification for the entire considered segment, i.e., calculate $g_{2}^{\prime }\left(t\right)=M_{2}\left(\left\{W_{k}^{p}\left(t\right)\right\}\right)$. For that purpose, we count classifications $g_{3}^{\prime }\left(t\right)=$’female’ and $g_{3}^{\prime }\left(t\right)=$’male’ delivered by $M_{3}$ for all vectors $X^{p}\left(t\right)\in \left\{W_{k}^{p}\left(t\right)\right\}$. The greater value, i.e., the value that better supports a particular gender, is selected as the classification of the entire segment, i.e., $g_{2}^{\prime }\left(t\right)=\arg\;max_{g}\;\alpha \left(\left\{W_{k}^{p}\left(t\right)\right\},g\right)$, where $\alpha (\left\{W_{n}^{p}\left(t\right),g\right\}$ is calculated as:

(1) $$\alpha \left(\left\{W_{n}^{p}\left(t\right)\right\},g\right)=\frac{|M_{3}\left(X^{p}\left(t\right)\right)=g|}{\left|\{W_{k}^{p}\left(t\right)\}\right|}.$$

In Formula (1), we denote by $|M_{3}\left(X^{p}\left(t\right)\right)=g|$ the number of correctly classified vectors $X^{p}\left(t\right)$ belonging to segment $\left\{W_{n}^{p}\left(t\right)\right\}$. The number of all vectors that are classified, i.e., the length of segment $\left\{W_{k}^{p}\left(t\right)\right\}$ is denoted by $\left|\{W_{k}^{p}\left(t\right)\}\right|$. Thus the value of α is the rate of correct classification yielded by $M_{3}$ for a particular gender *g* in particular *k*th segment $\left\{W_{k}^{p}\left(t\right)\right\}$.

Following backward our hierarchy of classifiers, we calculate the classification of the entire time series and, i.e., produce $g_{1}^{\prime }=M_{1}\left(\left\{X^{p}\left(t\right)\right)\right\}$. The issue is that $g_{2}^{\prime }\left(t\right)$ produced previously by $M_{2}$ is still time-dependent, i.e., it depends on the segment $W_{k}^{p}\left(t\right)$ for which it was obtained. For that reason, considering the entire $\left\{X^{p}\left(t\right)\right\}$ we get a series of $g_{2}^{\prime }\left(t\right)$, where some of them can be correct, i.e., $g_{2}^{\prime }\left(t\right)=g$, and some not, i.e., $g_{2}^{\prime }\left(t\right)\neq g$.

To cope with that issue, we must select the segment (the value of *k*) that will be used for gender recognition. For that purpose, we perform a series of computational experiments using the gathered data sample *S*. The goal of those experiments is to select that segment for which the classification $g_{2}^{\prime }\left(t\right)=M_{2}(\left\{\left(W_{k}^{p}\left(t\right)\right\}\right)$ is the best, i.e., it delivers the highest number of correctly identified genders. For each segment $\left\{W_{k}^{p}\left(t\right)\right\}$, $k=1,2,\ldots ,k_{\max}$ we calculate:

(2) $$\gamma \left(\left\{W_{k}\left(t\right)\right\}\right)=\frac{\sum_{p=1}^{p_{max}}|M_{2}\left(\left\{\left(W_{k}^{p}\left(t\right)\right\}\right)=g\right|}{p_{max}},$$

where *p* is an index of persons and $p_{max}$ is the number of them.

The segment with the highest γ, i.e., $W_{\mathrm{best}}=arg\;max{\;}_{k}\gamma \left(\left\{W_{k}\left(t\right)\right\}\right)$ is selected as that carrying the most useful information regarding the final classification. Therefore, we assume the classification of the chosen segment as that for the entire time series, i.e., $M_{1}\left(\left\{X^{p}\left(t\right)\right\}\right)=M_{2}\left(\left\{W_{\mathrm{best}}\left(t\right)\right\}\right)$.

Going to the uppermost level of the classification chain, we assume the data pre-processing Φ was made in the best possible way and enables us to conclude that $M=M_{1}$. This way, we solve the initially stated problem of gender recognition.

## 4. Results and Discussion

### 4.1. Experimental Setup

At each level of the proposed approach, we deal with a number of parameters. The values of those parameters are provided in Table 1.

Due to the low number of participants (20) we opted to use the leave-one-out cross-validation, meaning we performed 20 validation trials. In each of them, we used data gathered from 19 participants for learning classifier *M*. A single person left in each trial was used for testing. This way, we tested our approach for each of the 20 participating subjects. We also avoided randomness, and its consequences occurring, by using standard k-fold cross-validation.

Averaging classification results over 20 learn-and-test trials we calculated α and γ using Formulas (1) and (2), respectively. All the obtained results were rounded to two decimal places.

As the selection of the best classifier (among hundreds of those that are available) is a complex research problem that has been addressed for years [45], we relied in that case on the recommendations provided in the literature [45]. For the comparative experiments, we assumed Near-Neighbor (k-NN), Naive Bayes (NB), Random Forrest (RF), and Support Vector Machine (SVM) classifiers as those frequently used for the classification of EEG signals. Note that the chosen classifiers rely on diverse theoretical grounds. Thus, we validate different approaches to the classification task.

The k-NN classifier assumes that similar data instances can be classified to the same class [46]. The classifier is called lazy because it does not learn any data model. It only stores training data. The classifier calculates the distance between the query example and each of the stored data instances from the training data set. The collection of the calculated distances is sorted from smallest to largest. Then, the class labels are picked from the first *k* entries of that list, where k is the parameter of the classifier. The mode of those labels is returned as the classification of the query example. For this study, we used the k-NN with the most popular Euclidean distance method.

A completely different approach represents the NB classifier that relies on counting data instances while ignoring similarities between them. The classifier uses the Bayesian formula to calculate the most probable class label for the classified data instance [47]. For that purpose, conditional probabilities of each possible class $y|x_{i}$ and for each feature $x_{i}$ are calculated. In the case of numerical attributes, it is assumed that those values are sampled from a Gaussian distribution. As the NB also assumes that the features are independent, i.e., a feature does not affect the other, the calculation of $P(y|x_{1},\ldots ,x_{n})$ can be easily performed using the Bayes formula and the multiplication of the obtained probabilities. The class *y* with the maximum $P(y|x_{1},\ldots ,x_{n})$ is assigned to the query data instance.

The RF classifier is an ensemble learning method that uses a chosen number of decision trees [48]. As the RF relies on decision trees, it takes into account both the similarity and statistical properties of data. For this paper, we checked various tree split criteria and selected the information gain ratio as the most promising in terms of the finally obtained classification accuracy. Each of the decision trees is based on a different set of data instances and a different random set of features of size $\sqrt{p}$, where *p* is the total number of attributes. The output of the RF is the class selected by most trees.

The SVM classifier also relies on the calculation of distances between data instances [49]. In that sense, it is similar to the k-NN. However, in the case of the SVM, the goal is to maximize the margin distance between data instances. The margin is understood as a gap between the hyperplanes separating data instances belonging to different classes, and the positions and orientations of those hyperplanes are determined by support vectors. By their proper adjustment, it is possible to maximize the margins of the SVM. As a result, data instances falling on either side of the hyperplanes are attributed to different classes. The issue here is that the separation of data using linear hyperplanes is frequently challenging. Therefore, the SVM uses the so-called “kernel trick” to transform the original data to a higher-dimensional space. A kernel is a function that maps the data to a higher dimension. After performing numerous trials, for the purpose of this paper, we decided to use a polynomial kernel.

The statistical analysis was performed using R (Version 3.6.1) and RStudio (Version 1.1.463, RStudio, Inc., Boston, MA, USA) to determine whether the obtained accuracies of the classifiers were significantly above chance. Since the data did not meet the criterion of normal distribution, a non-parametric one-sample Wilcoxon signed-rank test with Bonferroni correction for multiple comparisons was used. p<0.05 was regarded as significant.

### 4.2. Experimental Results

In a standard oddball experiment, several ERPs are generated, of which the most important is the P3b waveform, related to attentional processes. It is a broad positive peak, from around 300 to 750 ms, with maximal amplitude around 350–500 ms after the stimulus onset. Other waveforms (N1, P2, and N2) are negative (N1 and N2) and positive (P2) peaks, reflecting sensory and early attention processes, and are observed between 100 and 250 ms post-stimulus (Figure 3).

At the first step of the proposed approach, we performed a series of experiments to select the time segment for which the obtained classification accuracy is the best. Table 2 shows accuracies for each time segment for four analyzed classifiers: 3-NN, NB, RF, and SVM.

**Figure 4:**
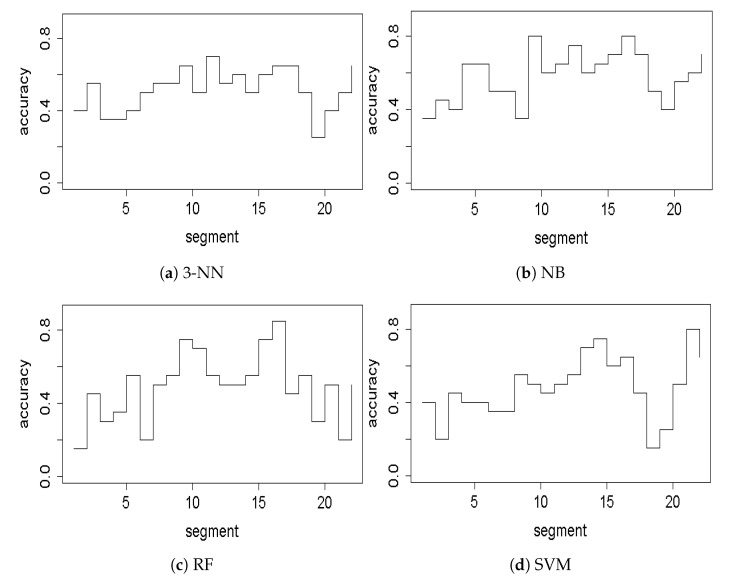
Classification accuracies for each analyzed classifier: (**a**) Near-Neighbor (kNN), (**b**) Naive Bayes (NB), (**c**) Random Forrest (RF), and (**d**) Support Vector Machine (SVM), plotted for all segments of the epoched ERP signal.

Since this study used a binary classification, we were interested in those accuracies that were above chance, i.e., 0.5. Moreover, due to their high temporal resolution, ERPs are the neural correlates of the dynamical cognitive processes occurring in the brain. Therefore, we can specify the time window for which the ERP signal is meaningful, i.e., it maps true brain electrical activity. Since the information in the visual modality in this paradigm is processed not earlier than after the first 100 ms, and in this experiment, we used an oddball paradigm to evoke the P3 waveform, which extends until around 750 ms, and we should expect meaningful results within 100–800 ms. All classifiers achieved high accuracy at the time segments for which statistical significance by means of non-parametric permutation cluster-based analysis was obtained in our previous experiment, i.e., approximately 540–710 ms [40], which corresponds to the segment numbers 14–16. Moreover, two of these classifiers achieved their maximal accuracies among all segments within this time window: γ=0.8 and 0.85 for NB and RF, respectively. Both of these accuracies were observed for segment 16 (650–700 ms). This shows an agreement of the classification performed in this study with the statistical analysis performed in our previous work. Interestingly, however, 3-NN, NB and RF classifiers also achieved high accuracies for segment 9 (300–350 ms), which corresponds to the time window when the P3 waveform rises and reaches the maximal amplitude. NB and RF classifiers were especially good because the performance of NB classifier reached its highest accuracy (γ=0.8) for this time segment among all segments, and the RF classifier achieved accuracy γ=0.75. To better visualize the high accuracies achieved within the neurobiologically interesting segments, the calculated accuracies are presented in Figure 4.

To summarize, we achieved successful gender classification based on the ERP waveform from the attention task. We obtained high accuracies in the neurobiologically meaningful time windows. In particular, the highest accuracies (0.8 and 0.85) were observed for segments, where a previous analysis revealed statistical differences between men and women, and for the additional segment, for which statistical analysis failed to find differences. This shows that our hierarchical classification approach is capable of recognizing the subject’s gender based on neurophysiological signal.

The next point was to compare the performance of the four studies classifier and to indicate the best one. The discussion of our results from the previous paragraph suggests 3-NN, NB, and RF might be good candidates. Therefore, we next compared the number of segments within the meaningful 100–800 ms time window, for which each classifier achieved accuracy above chance. As it can be seen in Figure 4, among 14 segments within this time window, the number of segments, for which γ>0.5 was: 9, 10, 9, and 6 for 3-NN, NB, RF, and SVM classifier, respectively.

Moreover, the fact that classification was highly accurate, especially for two segments discussed above (9 and 16) is interesting, because it shows an agreement with previous work (later time segment), but in addition, it also suggests there are gender-related differences in the earlier time window of the P3 waveform, which were not observed by statistical analysis, even using an advanced calculation method. Therefore, to further explore the classification performance within those two segments (given in columns of the table), we present in Table 3 and Table 4 the detailed results for each experimentation trial of the leave-one-out cross validation, for each used classifier. The number of trial corresponds to the person *p* for who the testing was performed.

**Figure 5:**
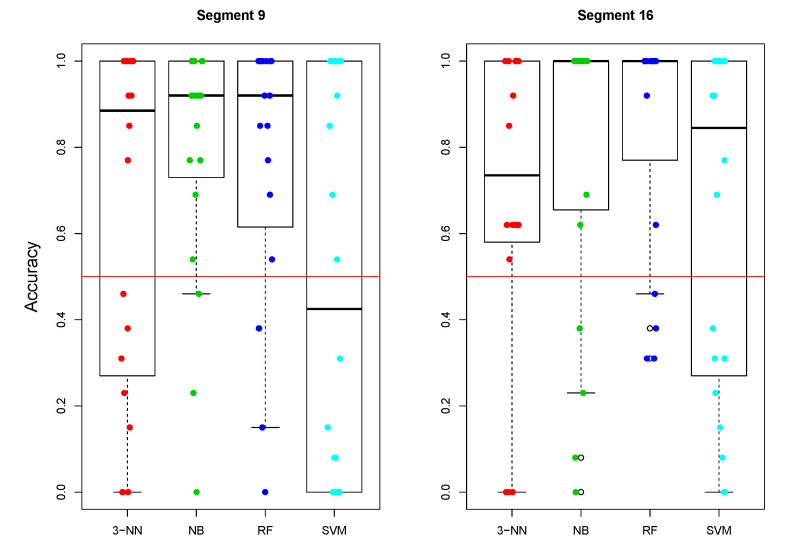
A distribution of classification rates (γ) for segment 9 (300–350 ms) and segment 16 (600–650 ms). The red horizontal line shows the chance level (0.5).

Having detailed information about the performance of each classifier for each participant, we checked whether the accuracies of the analyzed classifiers are above chance (Figure 5).

The statistical analysis revealed that an average accuracy for both segments: 9 (300–350 ms) and segment 16 (600–650 ms) was above chance only for NB ($V_{19}=187,\mathrm{Pcorr}=0.009$ and $V_{19}=187,\mathrm{Pcorr}=0.006$ for segments 9 and 16, respectively) and RF (($V_{19}=183$, Pcorr=0.014 and $V_{19}=198,\mathrm{Pcorr}=0.001$ for segments 9 and 16, respectively) classifiers, while not significantly different from the chance level for 3-NN (($V_{19}=148,\mathrm{Pcorr}=0.422$ and $V_{19}=152,\mathrm{Pcorr}=0.298$ for segments 9 and 16, respectively) and SVM (($V_{19}=103$, Pcorr=1.000 and $V_{19}=151,\mathrm{Pcorr}=0.353$ for segments 9 and 16, respectively) classifiers.

We also calculated the proportion of the participants, for which the accuracy of each classifier was above chance (>0.5) for segment 9 (300–350 ms) and 16 (650–700 ms). These proportions were: 0.6, 0.85, 0.8, and 0.5 for 3-NN, NB, RF, and SVM, respectively within segment 9, and: 0.8, 0.8, 0.8, and 0.6 for 3-NN, NB, RF, and SVM, respectively within segment 16. From these analyses and the comparison of the highest accuracies reached by these classifiers, discussed earlier, we can see that 3-NN, NB, and RF seem to perform much better than SVM and that NB and RF seem to be the best ones.

**Figure 6:**
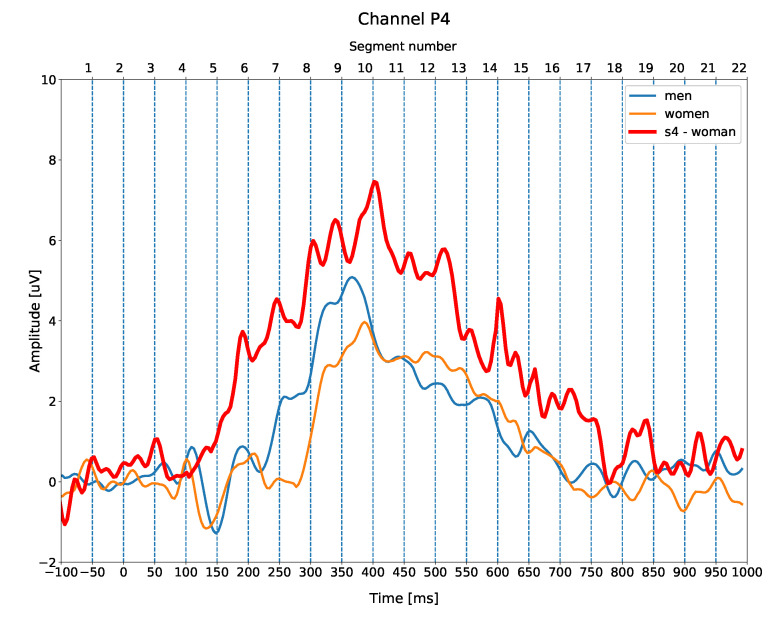
Individual ERP difference waveform from subject s4 (red) compared with averaged ERP difference waveforms for men (blue) women (orange) at electrode site P4 (right parietal).

As it can be seen in Table 3 and Table 4, the classification rate (γ) for a few participants was 0.0, i.e., the signal was not classified correctly for these segments. This means that individual ERP differences for these participants were more similar to the ERP differences in the opposite gender class. One of the reasons may be the nature of the ERP signal, i.e., the fact that it is an average of all trials measured time-locked to all of the presented stimuli. Therefore, variation from trial to trial of the single-trial waveform introduces distortion to the averaged waveform. The variability may also reflect stable individual differences among the subjects, e.g., due to differences in the pattern of individual folding of the cortex or neural processes. As an example, we present individual ERP of subject s4, who had the largest number of γ=0.0 (three out of eight) among both segments and all four classifiers. Figure 6 shows an individual ERP difference waveform from subject s4, compared with grand averaged ERP differences for men (blue) and women (orange).

The amplitude of the ERP trace for this subject is higher and noisier than both the grand-averages, for a simple reason that the grand average is the average of the voltage values of all participants, which are higher for some of them and lower for others. Therefore the grand averages are usually smaller than the most single-subject ERPs. These individual differences may cause the fact that an ERP falls closer to the opposite gender class for some individuals. Large inter- and intra- variability due to individual differences is a common issue in all studies performed on humans. However, our results show that even with a sample size as small as 20, we were able to obtain high gender classification accuracies.

To summarize, researchers recently have focused on developing new classification methods to improve neurophysiological signal analysis during rest and cognitive processes in normal and pathological conditions. However, decoding physiological signals is not trivial, especially among healthy participants, because differences in normal cognitive processes are less distinctive than differences between normal and pathological signals. One of the most intriguing attempts is gender recognition. Most gender recognition methods found in the literature focused on the external physiological features, where the recognition accuracy was poor, and classification efficiency was affected by feature extractors and classification algorithms [20]. A neurophysiological signal is a good alternative due to gender-related anatomical and/or functional differences. Unfortunately, due to large individual differences and the small effect size of gender differences, there is still a lack of powerful and sensitive methods. Some of the previous studies used EEG signal for gender classification [19,20,21,22,23,24,25,26,27]. However, these studies were based on resting-state EEG recordings, which do not capture gender-related differences in cognitive processes, such as attention. Two other studies [37,38] examined the EEG-based gender recognition related to emotions. However, to the best of our knowledge, there are no findings on gender-recognition method based on ERPs in attention.

Therefore, in this paper, we present a hierarchical approach for gender classification based on ERP signal collected during an attention task. This approach consisted of four-step-process, which covered data acquisition, data pre-processing, time series segmentation, and bottom level classification. One of the most important steps was data segmentation, which allowed us to divide the whole ERP epoch into smaller time segments. The highest accuracies obtained by the classifiers were observed for two segments within the timing of P3 waveform, being a neuronal correlated of attention: 9, which corresponded to 300–350 ms time interval, and 16, which corresponded to 650–700 ms time interval. Our previous work, which focused on understanding the underlying neural processes of attention, indicated possible gender-related differences in the speed and character of the P3 waveform. The effect presented here was observed as higher ERP amplitude in men than in women in the earlier time window (300–350 ms) and higher ERP amplitude in women than in men in the later time window (650–700 ms), which suggests that gender-related differences evoked in visual two-stimulus oddball paradigm are complex, and include changes in ERP waveforms generation, and distribution and suppression across the scalp, related to the attention process. The latter effect was in agreement with our previous statistical analysis, which showed significant differences between men and women within this time window. More interestingly, the classification rate was high also for the time window 300–350 ms, i.e., when the P3 waveform was rising and reaching the maximal amplitude. This shows the potential of our ERP-based method for gender classification. Moreover, comparisons of the performance of the most frequently used classifiers for the EEG signals (k-NN, NB, RF, and SVM) suggested the candidates that outperformed the others. In our future work, we are going to further explore the effectiveness of our approach for the classification of ERP signals.

## 5. Conclusions

In this paper, we proposed a new combined empirical and theoretical approach for an efficient gender recognition based on ERPs, in order to better understand neuronal differences between men’s and women’s brains for the attention task. We developed a new method for the hierarchical classification of ERPs, and broke the analysis into four steps: data acquisition, data pre-processing, time series segmentation, and bottom level classification. We ultimately formed a hierarchy of data processing steps, i.e., we formed a chain of classifiers $M\to M_{1}\to M_{2}\to M_{3}$, where each classifier is related to each step of the analysis process. Utilizing this, we transformed the addressed problem in a way that on its bottom level (classifier $M_{3}$) we were able to use a standard, state-of-the-art classifier known from the literature.

The proposed approach now enables us to recognize gender solely using the ERP signal measured in the attention task. As the experiments revealed, the proposed method is highly effective. It indicates gender differences in the attention task, which brings us additional knowledge on the differences between men and women in the functionality of the human brain during such an important cognitive process as attention. What is particularly important is that the classification approach not only correlated with the results obtained in our previous work, where a nonparametric permutation cluster-based analysis was used, but it also outperformed that analysis, and highlights the usefulness of ERP-based classification in electrophysiological signal analysis.

The main limitation of the proposed classification method is its dependency on the high number of parameters that have to be determined on each level of data processing and a small sample size. Further work is necessary towards the automatic adjustment of those parameters, as well as investigating other pre-processing and analysis approaches, e.g., ICA for artifact correction or comparing the results with repeated measures of ANOVA run on the 50 ms time bins.

This work has possible extensions to clinical studies and individual differences. Our focus on gender differences aimed at better understanding neuronal differences between men’s and women’s brains, as a fundamental factor in health and biomedical research at a basic level, but also in preclinical, clinical, and drug studies, which has far-reaching implications in reducing the misdiagnosis and adverse side effects in the under-represented women group. As a more sensitive method to capture gender-related differences in brain electrical activity, it may be a step towards minimizing the under-representation of women in the clinical studies, which in turn may result in smaller health risks, rates of misdiagnosis, and adverse side effects from drug treatment in women.

## Figures and Tables

**Table 1:** Parameters of the experiments.

Parameter	Description	Value
$p_{max}$	number of participants	20
*d*	number of electrodes	32
$n_{max}$	number of segments	22

**Table 2:** The values of $\gamma (\left\{W^{p}\left(t-1,t\right\}\right)$ parameters obtained for each analyzed classifier (Near-Neighbor: k-NN, Naive Bayes: NB, Random Forrest: RF, and Support Vector Machine: SVM), for each segment of the epoched ERP signal. The highest accuracy among all the segments of each classifier is marked in bold.

*k*	τ	3-NN	NB	RF	SVM
1	−100–50	0.4	0.35	0.15	0.4
2	−50–0	0.55	0.45	0.45	0.2
3	0–50	0.35	0.4	0.3	0.45
4	50–100	0.35	0.65	0.35	0.4
5	100–150	0.4	0.65	0.55	0.4
6	150–200	0.5	0.5	0.2	0.35
7	200–250	0.55	0.5	0.5	0.35
8	250–300	0.55	0.35	0.55	0.55
9	300–350	0.65	**0.8**	0.75	0.5
10	350–400	0.5	0.6	0.7	0.45
11	400–450	**0.7**	0.65	0.55	0.5
12	450–500	0.55	0.75	0.5	0.55
13	500–550	0.6	0.6	0.5	0.7
14	550–600	0.5	0.65	0.55	0.75
15	600–650	0.6	0.7	0.75	0.6
16	650–700	0.65	**0.8**	**0.85**	0.65
17	700–750	0.65	0.7	0.45	0.45
18	750–800	0.5	0.5	0.55	0.15
19	800–850	0.25	0.4	0.3	0.25
20	850–900	0.4	0.55	0.5	0.5
21	900–950	0.5	0.6	0.2	**0.8**
22	950–1000	0.65	0.7	0.5	0.65

**Table 3:** Classification rate (γ) for each experimentation trial of the leave-one-out cross validation, for each used classifier for segment 9 (300–350 [ms]). The number of the trial corresponds to the person *p* for who the testing was performed.

Person	3-NN	NB	RF	SVM
1	1.0	0.92	1.0	0.69
2	1.0	1.0	1.0	0.0
3	1.0	0.92	1.0	0.85
4	0.23	1.0	1.0	0.0
5	1.0	1.0	1.0	0.0
6	0.92	0.92	0.69	0.0
7	1.0	1.0	1.0	0.08
8	0.38	0.54	0.77	0.54
9	0.0	0.23	0.15	0.0
10	0.92	0.92	0.85	0.08
11	0.46	1.0	1.0	1.0
12	0.85	0.85	0.92	0.15
13	0.0	0.85	0.54	0.0
14	0.0	0.77	0.92	0.92
15	0.77	0.69	0.38	0.31
16	1.0	0.77	0.85	1.0
17	1.0	0.92	0.38	1.0
18	1.0	1.0	1.0	1.0
19	0.15	0.46	1.0	1.0
20	0.31	0.0	0.0	1.0

**Table 4:** Classification rate (γ) for each experimentation trial of the leave-one-out cross validation, for each used classifier for segment 16 (650–700 [ms]). The number of the trial corresponds to the person *p* for who the testing was performed.

Person	3-NN	NB	RF	SVM
1	0.92	1.0	1.0	0.92
2	0.85	1.0	1.0	0.69
3	0.62	0.23	0.31	0.0
4	0.0	0.38	0.62	0.0
5	1.0	1.0	1.0	0.23
6	0.62	0.0	0.31	0.38
7	0.54	0.69	0.46	0.31
8	1.0	1.0	1.0	1.0
9	1.0	1.0	1.0	1.0
10	0.62	1.0	1.0	0.92
11	0.62	1.0	1.0	0.08
12	0.0	1.0	1.0	1.0
13	0.62	1.0	1.0	0.77
14	1.0	1.0	1.0	1.0
15	1.0	1.0	1.0	1.0
16	1.0	1.0	1.0	1.0
17	1.0	0.62	0.92	0.31
18	1.0	1.0	1.0	1.0
19	0.0	1.0	1.0	1.0
20	0.0	0.08	0.38	0.15

## Data Availability

The data presented in this study are openly available in OSF.IO at https://osf.io/ef7uv/ (accessed on 19 November 2021), reference number [DOI 10.17605/OSF.IO/EF7UV].
